# HARNESSING TECHNOLOGY TO SUPPORT PERSONS WITH DEMENTIA AND THEIR CAREGIVERS

**DOI:** 10.1093/geroni/igz038.1429

**Published:** 2019-11-08

**Authors:** Stacy L Andersen, Walter Boot, Jeffrey Kaye

**Affiliations:** 1 Boston University School of Medicine, Boston, Massachusetts, United States; 2 Florida State University, Tallahassee, Florida, United States; 3 Oregon Health & Science University, Portland, Oregon, United States

## Abstract

One in eight older adults in the US has Alzheimer’s disease or a related dementia, which are characterized by progressive cognitive and physical declines. The impact of dementia also goes beyond the individual since 92% of persons with dementia receive functional and emotional support from family members and other informal caregivers. The time demands, financial strain, and emotional toll of caregiving are known to cause increased stress and health problems. Therefore, there is a wealth of opportunities to develop new ways to intervene in the progressive loss of function among persons with dementia and ways to support them and their caregivers. Co-sponsored by the Alzheimer’s Disease and Related Dementias and Technology and Aging Interest Groups, this symposium addresses innovations in the implementation of new and existing technologies in the dementia care continuum. We will discuss the development and testing of a new mobile application designed to integrate both physical activity and cognitive training. Then we will discuss results from a virtual support group intervention to provide disease education, care planning, and emotional and social support among persons newly diagnosed with Alzheimer’s disease and living alone. Next we will share results from a study using customized voice-assisted technologies to enable individuals with memory impairment to maintain independence and quality of life and reduce caregiver burden. Finally, we will present findings regarding the validity and accuracy of a wearable sensor-based device that measures skin conductivity and heart rate variability to monitor stress level among caregivers of persons with dementia.

